# Borneol Is a TRPM8 Agonist that Increases Ocular Surface Wetness

**DOI:** 10.1371/journal.pone.0158868

**Published:** 2016-07-22

**Authors:** Gui-Lan Chen, Ming Lei, Lu-Ping Zhou, Bo Zeng, Fangdong Zou

**Affiliations:** 1 Ministry of Education Key Laboratory of Bio-resources and Eco-environment, College of Life Sciences, Sichuan University, Chengdu, China; 2 Key Laboratory of Medical Electrophysiology, Ministry of Education, and Institute of Cardiovascular Research, Sichuan Medical University, Luzhou, China; University of Hull, UNITED KINGDOM

## Abstract

Borneol is a compound widely used in ophthalmic preparations in China. Little is known about its exact role in treating eye diseases. Here we report that transient receptor potential melastatin 8 (TRPM8) channel is a pharmacological target of borneol and mediates its therapeutic effect in the eyes. Ca^2+^ measurement and electrophysiological recordings revealed that borneol activated TRPM8 channel in a temperature- and dose-dependent manner, which was similar to but less effective than the action of menthol, an established TRPM8 agonist. Borneol significantly increased tear production in guinea pigs without evoking nociceptive responses at 25°C, but failed to induce tear secretion at 35°C. In contrast, menthol evoked tearing response at both 25 and 35°C. TRPM8 channel blockers N-(3-Aminopropyl)-2-[(3-methylphenyl)methoxy]-N-(2-thienylmethyl)benzamide hydrochloride (AMTB) and N-(4-*tert*-butylphenyl)-4-(3-chloropyridin-2-yl)piperazine-1-carboxamide (BCTC) abolished borneol- and menthol-induced tear secretion. Borneol at micromolar concentrations did not affect the viability of human corneal epithelial cells. We conclude that borneol can activate the cold-sensing TRPM8 channel and modestly increase ocular surface wetness, which suggests it is an active compound in ophthalmic preparations and particularly useful in treating dry eye syndrome.

## Introduction

Borneol is a bicyclic monoterpenoid compound extracted from medicinal plants such as *Blumea balsamifera* [1] and *Dryobalanops aromatica* [2], or synthesized by chemical reactions [3]. In China, borneol is widely used in ophthalmic preparations and classic formulas of Traditional Chinese Medicine to treat various diseases including oculopathies, oral ulcers, sore throat, skin diseases and mild neurological disorders [4], and found to produce anti-inflammatory, anti-bacterial and analgesic effects [5]. Borneol-containing eye drops account for a large proportion of over-the-counter (OTC) eye drops sold in China. It was reported that borneol can promote the penetration of various drugs through the cornea, including puerarin, timolol maleate [6], indomethacin, dexamethasone [7], fluconazole [8] and geniposide [9]. This effect was confirmed by using fluorophores with different hydrophilicities and molecular sizes [10]. Apart from this, little is known about the effects of borneol in the eyes.

Transient receptor potential melastatin 8 (TRPM8) is a non-selective cation channel belonging to the TRP channel family. The most important physiological function of TRPM8 discovered so far is its role in peripheral sensory neurons as the cold thermoreceptor [11]. Activation of TRPM8 channels by cold temperatures (below 22–26°C) leads to depolarization of membrane potential and initiation of action potentials in neurons [12–14]. In addition to cold temperatures, TRPM8 can also be activated by cool sensation-producing compounds such as menthol and icilin [15]. Previous studies have shown that TRPM8 channels are present in the corneal afferent neurons and mediate tear production induced by cooling ocular surface temperature or corneal application of menthol [16, 17]. Ca^2+^ influx through TRPM8 channels triggers action potential in the afferent neurons and transduction of cold stimuli in the neural circuit [18, 19], which consequently results in reflex tearing response [20, 21].

Dry eyes occur when the lachrymal glands are unable to secret sufficient tears to maintain a healthy coating on the corneal surface. If the dry eye condition becomes chronic and progressive, and is associated with uncomfortable feelings like sting, burn or itch, it is termed as dry eye syndrome [22]. Dysfunction of corneal cold thermoreceptors has been found to contribute to the development of dry eye disease [23]. As borneol-containing eye drops can elicit a cooling sensation when applied to the corneal surface [24], and application of borneol directly on the eyes or in ultrasonic cool mist therapy has been shown to improve basal tearing in dry eye patients [25, 26], we hypothesized that it may modulate corneal cold thermoreceptor directly and hence regulate ocular surface wetness. In the present study we examined the action of borneol on TRPM8 channels and investigated its effect on tear production in guinea pigs.

## Materials and Methods

### Chemicals

General salts, (+)-borneol and (-)-menthol were purchased from Sangon (Shanghai, China). N-(3-Aminopropyl)-2-[(3-methylphenyl)methoxy]-N-(2-thienylmethyl)benzamide hydrochloride (AMTB) was purchased from Santa Cruz Biotechnology (Dallas, USA). N-(4-*tert*-butylphenyl)-4-(3-chloropyridin-2-yl)piperazine-1-carboxamide (BCTC) and Fura-2/AM were purchased from Sigma-Aldrich (Shanghai, China).

### Cell culture, transfection and viability assay

Human embryonic kidney (HEK) 293 and primary human corneal epithelial (HCE) cells were purchased from ATCC (Manassas, USA), cultured in D-MEM/F-12 medium (HyClone, Shanghai, China) supplemented with 10% fetal bovine serum, 100 units/ml penicillin and 100 μg/ml streptomycin, and maintained at 37°C under 95% air and 5% CO_2_. The coding sequence of human TRPM8 gene (NM_024080) was synthesized by FulenGen (Guangzhou, China) and cloned into the pcDNA3.1(+) vector. The pcDNA3.1-TRPM8 plasmids were transfected into HEK293 cells with Lipofectamine 2000 (Invitrogen, Shanghai, China). The stably transfected cells were selected with 400 μg/ml G418 in the cell culture medium for 2 weeks and functionally characterized by patch-clamp recordings. The cells with resistance to G418 and TRPM8 currents were cultured for long-term use.

The viability of HEK293 and HCE cells was measured with WST-1 reagent (Roche, Shanghai, China) according to the manufacture’s instruction. Briefly, cells were seeded in 96-well plates to reach a confluency of 50–60%. Borneol or menthol was added into the cell culture medium and incubated for 24 h. WST-1 (10 μl) was then added into each well and incubated for 2 h. Absorbance at 450 nm (A_450_) and 690 nm (A_690_) was measured in a plate reader (Tecan, Switzerland). The subtracted value (A_450_-A_690_) was used to evaluate viability of the cells.

### Ca^2+^ measurement

The TRPM8-expressing HEK293 cells were seeded on 13-mm glass coverslips and cultured for 24–48 h. Before the measurement cells were loaded with 2 μM Fura-2 AM in standard bath solution for 30 min at 37°C, and then washed for 5 min with standard bath solution. Ca^2+^ measurement was performed in a heating chamber to maintain solution temperature at 25 or 35°C. Cells were excited alternately by 340- and 380-nm light, and emission was collected via a 510-nm filter. Images were sampled every 5 s in pairs for the two excitation wavelengths by a CCD camera. The ratio of 340/380 nm fluorescence was used to represent the intracellular Ca^2+^ level. The standard bath solution contained (mM): NaCl 130, KCl 5, MgCl_2_ 1.2, HEPES 10, Glucose 8, and CaCl_2_ 1.5 (pH 7.4). Ca^2+^-free solution contained (mM): NaCl 130, KCl 5, MgCl_2_ 1.2, HEPES 10, Glucose 8, and EGTA 0.4 (pH 7.4). The *n* values given are the numbers of cells from at least three independent Ca^2+^ imaging experiments.

### Electrophysiology

Patch-clamp recordings were performed in a heating chamber to maintain solution temperature at 25 or 35°C. The signal was amplified with an Axon CNS MultiClamp 700B or a HEKA EPC10 USB amplifier controlled by the software pClamp 10.6 or Patchmaster 2.90, respectively. In whole-cell recordings, the standard bath solution in Ca^2+^ measurement was used as external solution. The resistances of glass microelectrodes were 3–5 MΩ when filled with 200 nM Ca^2+^ buffered pipette solution: 115 CsCl, 10 EGTA, 2 MgCl_2_, 10 HEPES, and 5.7 CaCl_2_ (in mM, pH 7.2 adjusted with CsOH, and osmolarity ~290 mOsm adjusted with mannitol). The sampling rate was 4 kHz. A 1-s ramp voltage protocol from –100 mV to +100 mV was applied at a frequency of 0.2 Hz from a holding potential of 0 mV. The junction potential between the intracellular and extracellular solution, and fast and slow capacitance transients were compensated by the software of the amplifier. Series resistance (R_s_) was compensated up to 51% and the access resistances were limited to <10 MΩ to reduce voltage errors. Membrane capacitance was calculated by the software and the mean value was 13.90±3.41 pF (*n* = 45). Current recordings with seal resistance close to or over 1 GΩ were used for analyses after leak-subtraction (leak currents <100 pA). For inside-out patches, the bath solution contained (in mM) 140 KCl, 10 HEPES, and 1 EGTA (pH 7.2 adjusted with KOH). The pipettes were 15–20 MΩ and filled with symmetrical high K^+^ solution (in mM): 140 KCl, 10 HEPES, 1 EGTA (pH 7.4 adjusted with KOH). The signals were sampled at 10 kHz and digitally filtered off-line at 0.8 kHz. The sampling time of each inside-out recording was 25 s and the holding potential was +100 mV.

### Animal experiment

Guinea pigs of both sexes, aged 5–7 weeks and in the weight range of 200–250 g were obtained from the experimental animal facility of Sichuan Medical University. The animals were housed in plastic cages at room temperature (25–26°C), fed with commercial pellet diet and tap water, and subjected to natural light/dark cycles. Guinea pigs were monitored daily for normal behaviors, regular food/water intake and clean furs, which were considered as signs of general health. No animals became ill throughout the experiment. All the animal experiment was performed at environmental temperature of 25–26°C. For tear measurement, the animals were restrained on a perforated plate with four limbs loosely tied on the plate. Guinea pigs could move the limbs within a restricted range (2–3 cm), but were unable to touch the eyes and wipe off the tears with paws. The duration of restraining on the plate was no more than 10 min for each animal. None of the animals were inadvertently injured as a result of being restrained. As tear secretion involves a neurological response, no anesthetic procedure was used prior the experiment. After a 5-min acclimation on the plate, 10 μl prewarmed (25°C or 35°C) physiological saline solution containing borneol, menthol or vehicle (0.1% DMSO) were dropped into each eye with a micropipette. Channel blockers AMTB and BCTC were combined with borneol or menthol and applied to the eyes in a same way. After 2 min, a phenol red thread with diameter of ~0.5 mm and length of 75 mm (Jingming, Tianjin, China) was placed in the inferior conjunctival sac of the eye for 15 s. The length of color-changed part of the thread was measured under a microscope immediately after removal from the eye. Nociceptive responses of freely moving animals were measured by recording the times and duration of eye closure, eye wiping with paws and blink within 5 min after the application of saline, vehicle, borneol or menthol solution at room temperature. Guinea pigs were sacrificed by intraperitoneal injection of sodium pentobarbital (100 mg/kg) after the experiment, and exsanguination was performed 30 min later. Corneas were then dissected from the eye balls for further use. All the procedures above were approved by the Ethical Committee of Sichuan Medical University (Permit Number: KY2013-024) and meet internationally accepted principles for the use of experimental animals.

### Reverse transcription-polymerase chain reaction (RT-PCR)

Total RNA was extracted from corneas of guinea pigs, primary HCE cells, TRPM8-transfected and control HEK293 cells using an RNAprep Pure Tissue Kit (TIANGEN, Beijing, China). First-strand cDNA was synthesized with a ReverTra Ace -α- Kit (TOYOBO, Shanghai, China). A 259-bp TRPM8 fragment was amplified from the cDNA with One*Taq* Quick-Load 2X Master Mix (New England BioLabs, Beijing, China) and following primers: gpTrpm8-F, TCCGTCTGTCCTGCGATAC and gpTrpm8-R, TTCCTGCTGATGGTGTTGTC (synthesized by Sangon, Shanghai, China). PCR program consisted of an initial denaturation at 95°C for 2 min, and 35 cycles of 95°C 20 s, 56°C 20 s and 72°C 20 s. PCR product was analyzed in 1.5% agarose gel and sequenced.

### Statistics

All values are expressed as mean ± S.E.M.. Unpaired Student’s *t* test was used to assess the statistical difference between two groups. One way ANOVA was used in comparison of more than two groups. *P* value less than 0.05 was considered as significant.

## Results

### Borneol evokes Ca^2+^ influx through TRPM8 channel

**Fig 1:**
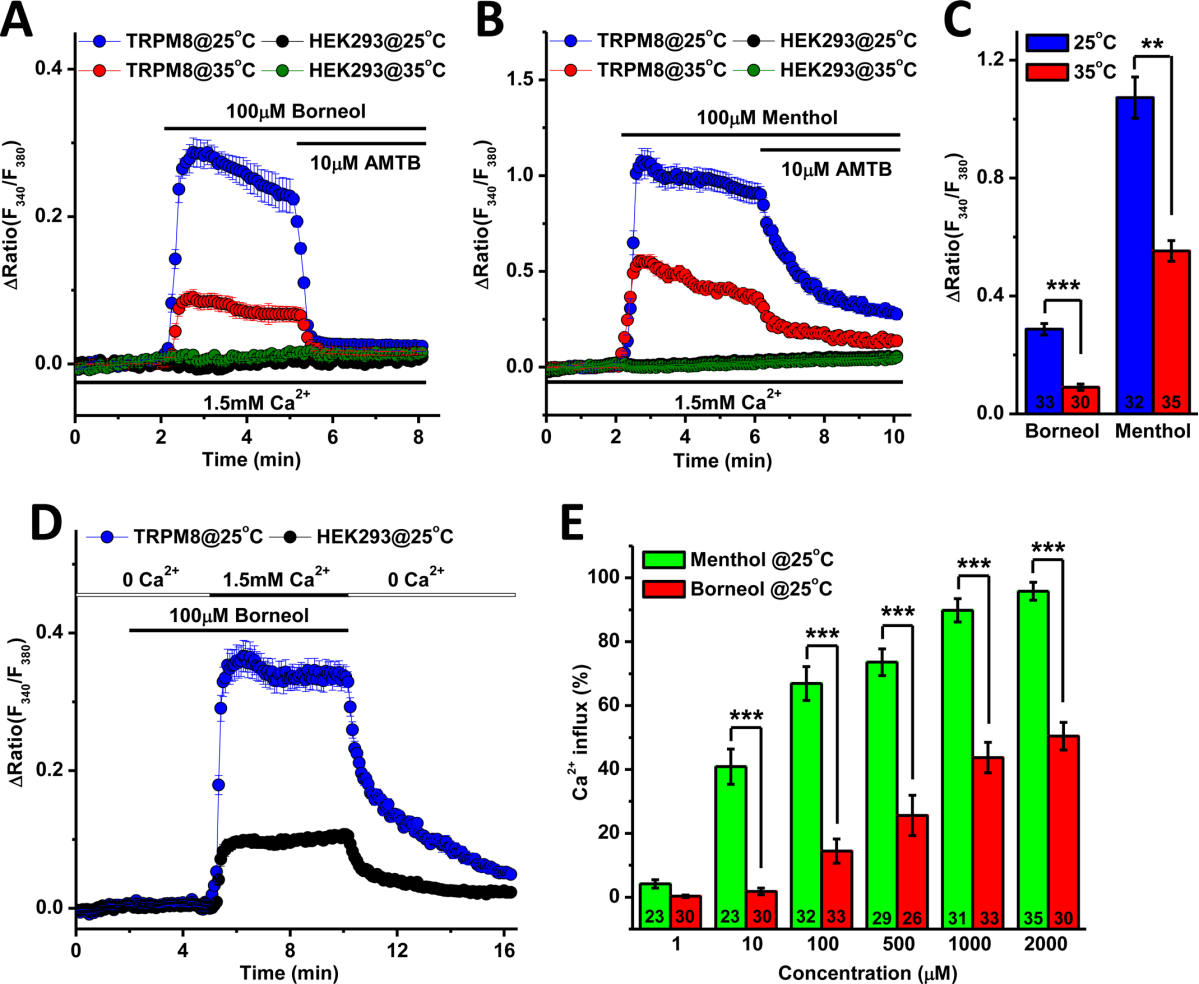
Borneol stimulated Ca^2+^ influx in HEK293 cells overexpressing TRPM8 channel. (A-B) In the presence of 1.5 mM extracellular Ca^2+^, borneol and menthol induced an increase of cytosolic Ca^2+^ in TRPM8-expressing cells, but not in the control HEK293 cells without TRPM8 expression at 25 and 35°C. The rise of Ca^2+^ was inhibited by 10 μM AMTB, a selective TRPM8 blocker. (C) Efficacy of 100 μM borneol and menthol on inducing Ca^2+^ influx into TRPM8-expressing cells at 25 and 35°C. (D) In the absence of extracellular Ca^2+^, borneol did not change the intracellular Ca^2+^ level, while subsequent addition of extracellular Ca^2+^ resulted in robust Ca^2+^ influx. (E) Comparison between the effects of borneol and menthol at different concentrations on the induction of Ca^2+^ influx in TRPM8-expressing cells. ** *P*<0.01, *** *P*<0.001. Sample numbers are shown in the bars.

We generated a HEK293 cell line stably overexpressing human TRPM8 channel to test the channel sensitivity to borneol. The expression of TRPM8 in this cell line was confirmed by Western blotting and RT-PCR (S1 Fig). As shown in Fig 1A, borneol (100 μM) induced Ca^2+^ influx in TRPM8-expressing cells at 25°C and 35°C, but not in the control HEK293 cells without TRPM8 expression. Borneol-induced Ca^2+^ influx was abolished by 10 μM AMTB (Fig 1A), a selective blocker of TRPM8 channel [27, 28]. BCTC (20 μM), another blocker with less selectivity for TRPM8, showed similar inhibitory effect (S2 Fig). These results suggest that TRPM8 channel is a pharmacological target of borneol. As a positive control, menthol (100 μM) potently stimulated Ca^2+^ entry into TRPM8-expressing cells, which was also inhibited by AMTB (Fig 1B). No significant effect of menthol was observed on control HEK293 cells. For both borneol and menthol, the levels of Ca^2+^ influx at 25°C were significantly higher than that at 35°C (Fig 1C). In addition, borneol (100 μM) failed to stimulate intracellular Ca^2+^ rise when the TRPM8-expressing cells were kept in Ca^2+^-free solution (Fig 1D), suggesting that the increase of intracellular Ca^2+^ level upon Ca^2+^ admission is due to TRPM8-mediated Ca^2+^ influx from extracellular space, but not Ca^2+^ release from intracellular stores, such as endoplasmic reticulum and mitochondria. We also compared the potency of borneol and menthol on the activation of TRPM8 channel at 25°C by Ca^2+^ measurement. The result showed that borneol was much less effective than menthol at the same concentration within the range of 10 μM to 2 mM (Fig 1E).

### Borneol activates TRPM8 currents

**Fig 2:**
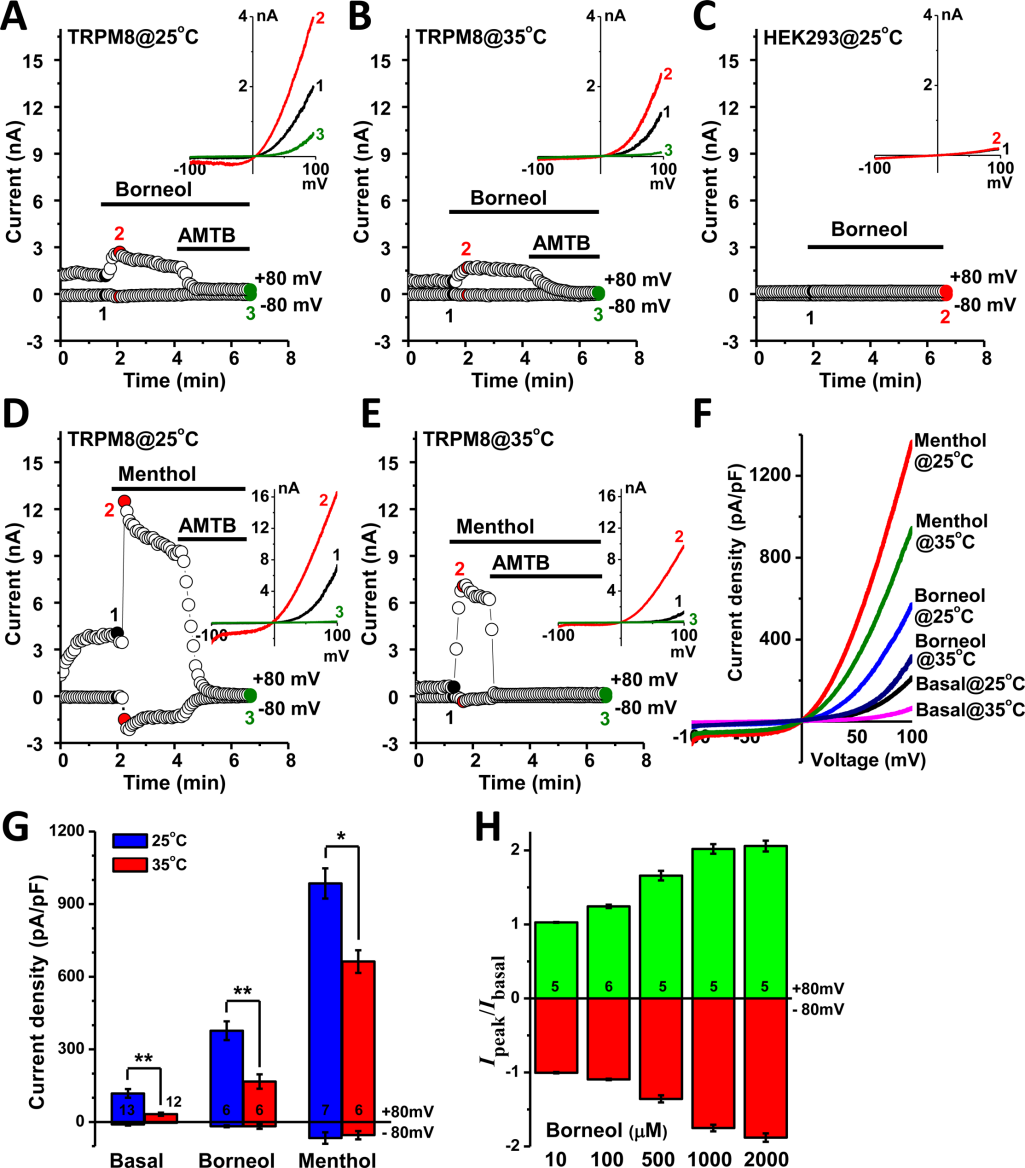
Borneol potentiated TRPM8 currents in transfected HEK293 cells. (A-B) Acute stimulation of whole-cell currents in TRPM8-expressing cells by 100 μM borneol at 25 and 35°C. Subsequent superfusion of 10 μM AMTB potently inhibited the currents. The inset shows current-voltage (*I-V*) relationships collected from the time points 1, 2 and 3. (C) Borneol (100 μM) showed no effect on the whole-cell currents in non-transfected HEK293 cells. (D-E) Menthol (100 μM) potently augmented the whole-cell currents in TRPM8-expressing cells at 25 and 35°C. (F) Traces for the mean values of whole-cell current density-voltage relationships at conditions indicated. (G) Statistical results for values extracted from ±80mV in panel F. Sample numbers are shown in the bars. (H) Dose-dependency of borneol on the activation of whole-cell TRPM8 currents at 25°C. The ratio between borneol-induced peak current and the basal current before borneol application was used.

**Fig 3:**
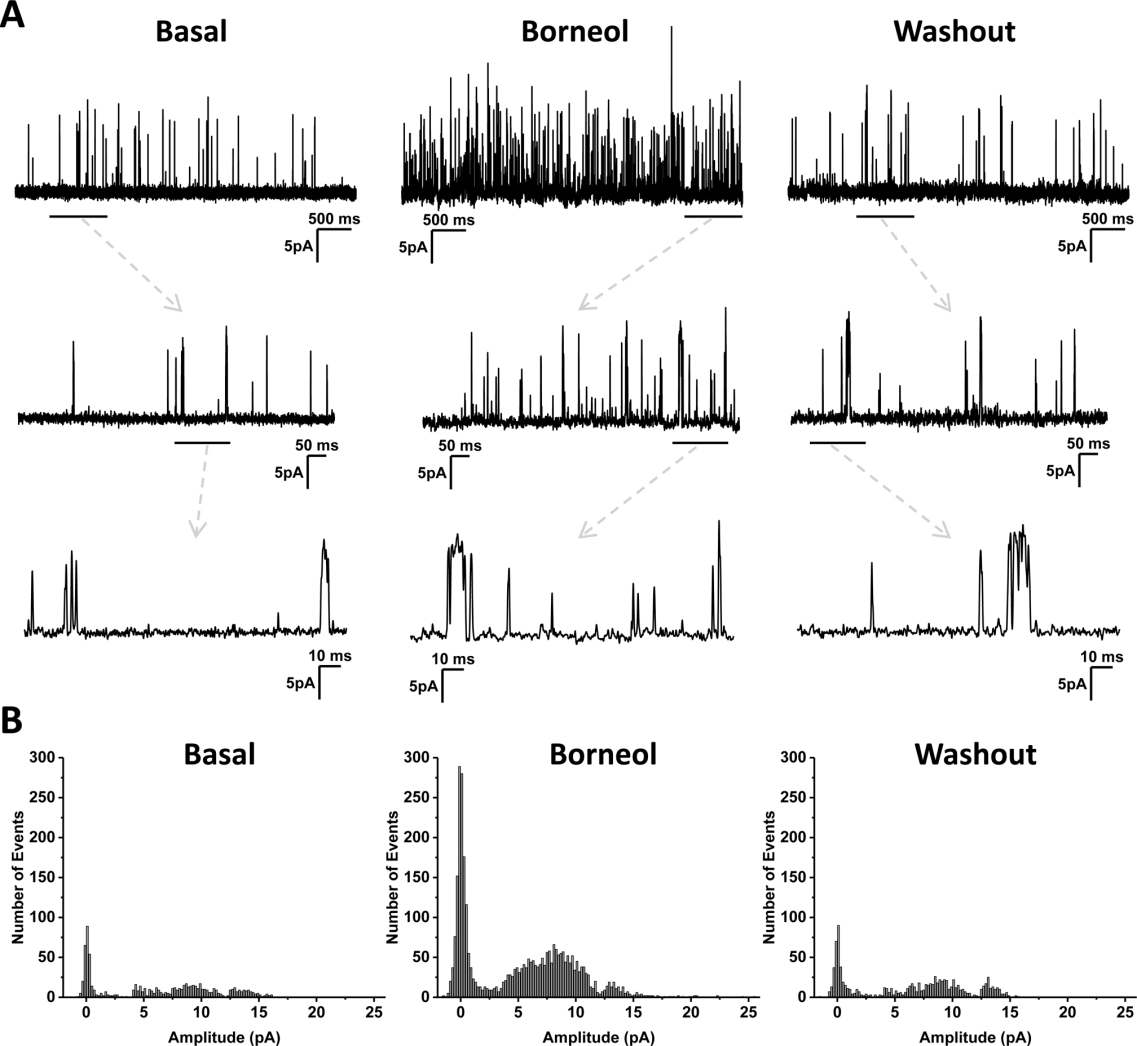
Effect of borneol on TRPM8 currents in transfected HEK293 cells recorded under inside-out configuration. (A) Typical recordings of single channel currents at +100 mV and 25°C. Borneol at 100 μM was used. Dashed arrows indicate expansions from underlined region above. (B) Amplitude histograms of the single channel activity. The histograms could not be fitted with a simple Gaussian function, reflecting the existence of multiple conducting states.

In whole-cell configuration, TRPM8 channels expressed in HEK293 cells exhibited some basal activities at 25°C before drug application (Fig 2). Superfusion of 100 μM borneol immediately increased the amplitudes of both inward and outward currents at 25 and 35°C, which were then completely abolished by 10 μM AMTB (Fig 2A and 2B) and 20 μM BCTC (S2 Fig). In consistent with the observation in Ca^2+^-imaging experiment, 100 μM borneol did not evoke any signal in control cells without TRPM8 expression (Fig 2C), which confirmed that TRPM8 channel was responsible for the stimulatory effect of borneol. The currents activated by 100 μM menthol were much larger and exhibited a gradual desensitization, and were fully inhibited by 10 μM AMTB (Fig 2D and 2E). The densities of whole-cell currents recorded at 25°C were significantly higher than that recorded at 35°C under the same condition, and menthol was found more potent than borneol at both temperatures (Fig 2F and 2G). The activation of TRPM8 channel by borneol was concentration-dependent within the range of 10 μM to 2 mM (Fig 2H). Inside-out patch-clamp recording revealed that application of borneol on the intracellular side of plasma membrane stimulated channel activities, and the currents decreased to the basal level after the drug was washed away (Fig 3A). The action of borneol was mainly attributed to the increase of channel opening probability (Fig 3B).

### Borneol stimulates lacrimation without evoking nociceptive responses in guinea pigs

**Fig 4:**
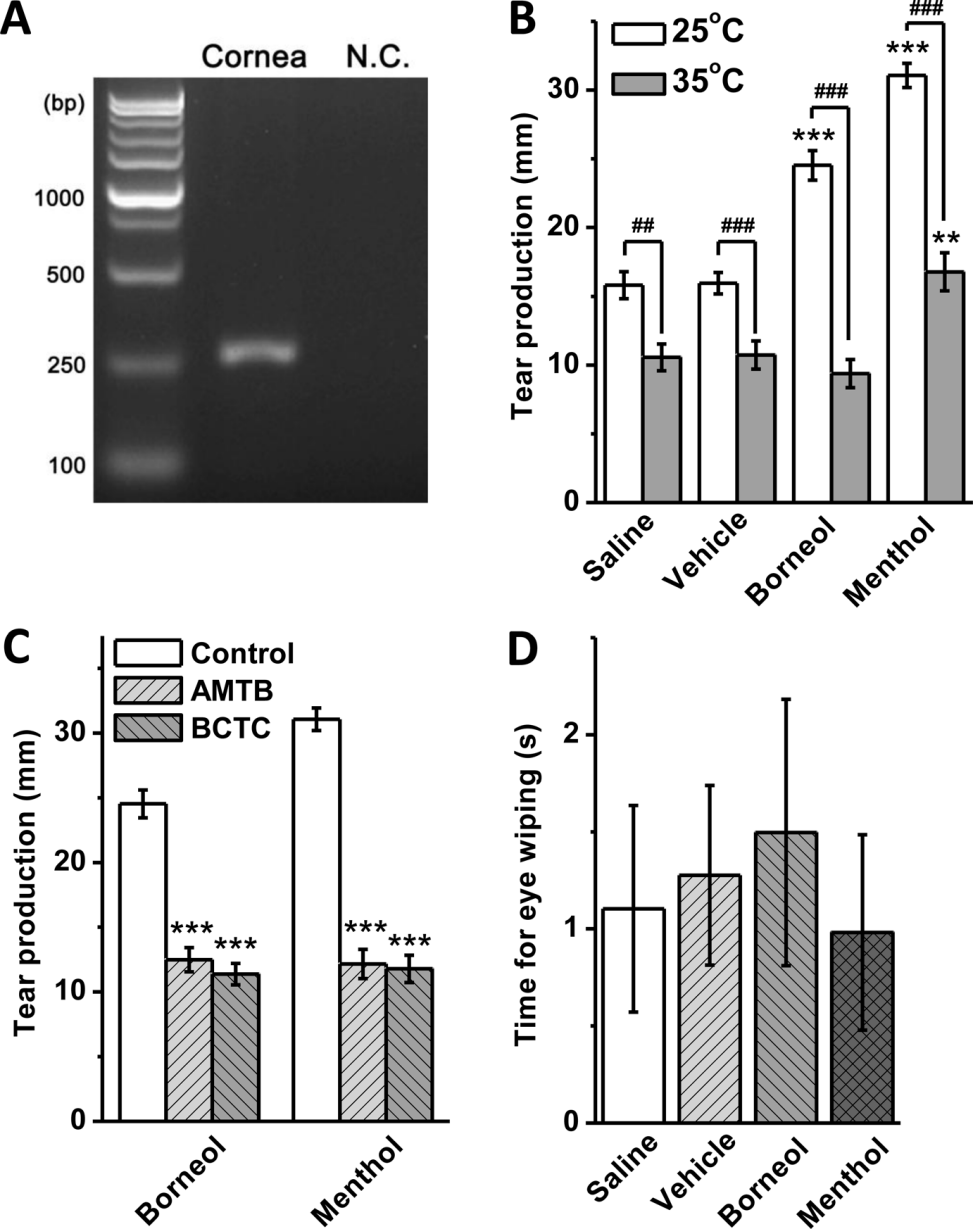
Borneol evoked tear secretion without nociceptive effect in guinea pigs. (A) RT-PCR of TRPM8 mRNA expressed in the cornea of guinea pig. N.C., negative control. (B) 100 μM borneol and menthol were applied to the ocular surface at 25 and 35°C to induce tear production (** *P*<0.01, *** *P*<0.001 *vs* vehicle at the same temperature; ^##^ *P*<0.01, ^###^ *P*<0.001 between groups indicated; *n* = 10 in each group). (C) TRPM8 channel blockers AMTB (10 μM) and BCTC (20 μM) inhibited borneol- and menthol-induced tear production (*** *P*<0.001 *vs* control; *n* = 10 in each group). (D) Total time spent for eye wiping with paws by the animals within 5 min after the drop of 100 μM borneol and menthol solution to the corneas. No significantly difference was observed between groups (*n* = 4 in each group).

Expression of TRPM8 transcripts in the cornea of guinea pig was detected by RT-PCR (Fig 4A) and confirmed by sequencing. In tear measurement, the animals started tearing soon after the solution was dropped to the ocular surface. The tearing process lasted about 5 min; however, the animals always shook away excessive tears after 2 min, making it difficult to collect all tears through the whole tearing process. To make an exact quantitative comparison, we collected tears from the canthi at 2 min after the drop of solution. At 25°C, both borneol and menthol at 100 μM increased tear secretion, which was significantly higher than that in the saline and vehicle groups (Fig 4B). When the solution was prewarmed to 35°C, the tear secretion was greatly reduced compared to the results at 25°C, and no significant difference was observed between borneol-treated and control groups at 35°C (Fig 4B). A significant increase of tear secretion at 35°C was observed in menthol-treated group; however it was still much lower than that induced by menthol at 25°C (Fig 4B). The application of TRPM8 channel blockers AMTB (10 μM) and BCTC (20 μM) abolished the stimulatory effect of borneol and menthol on tear production at 25°C (Fig 4C). In the test for nociceptive responses, eye closure and blink were not observed during the 5 min of drug application in all animals. Total time of eye wiping with paws within 5 min was comparable between the borneol, menthol, vehicle and saline groups (Fig 4D).

### The effects of borneol and menthol on cell viability

**Fig 5:**
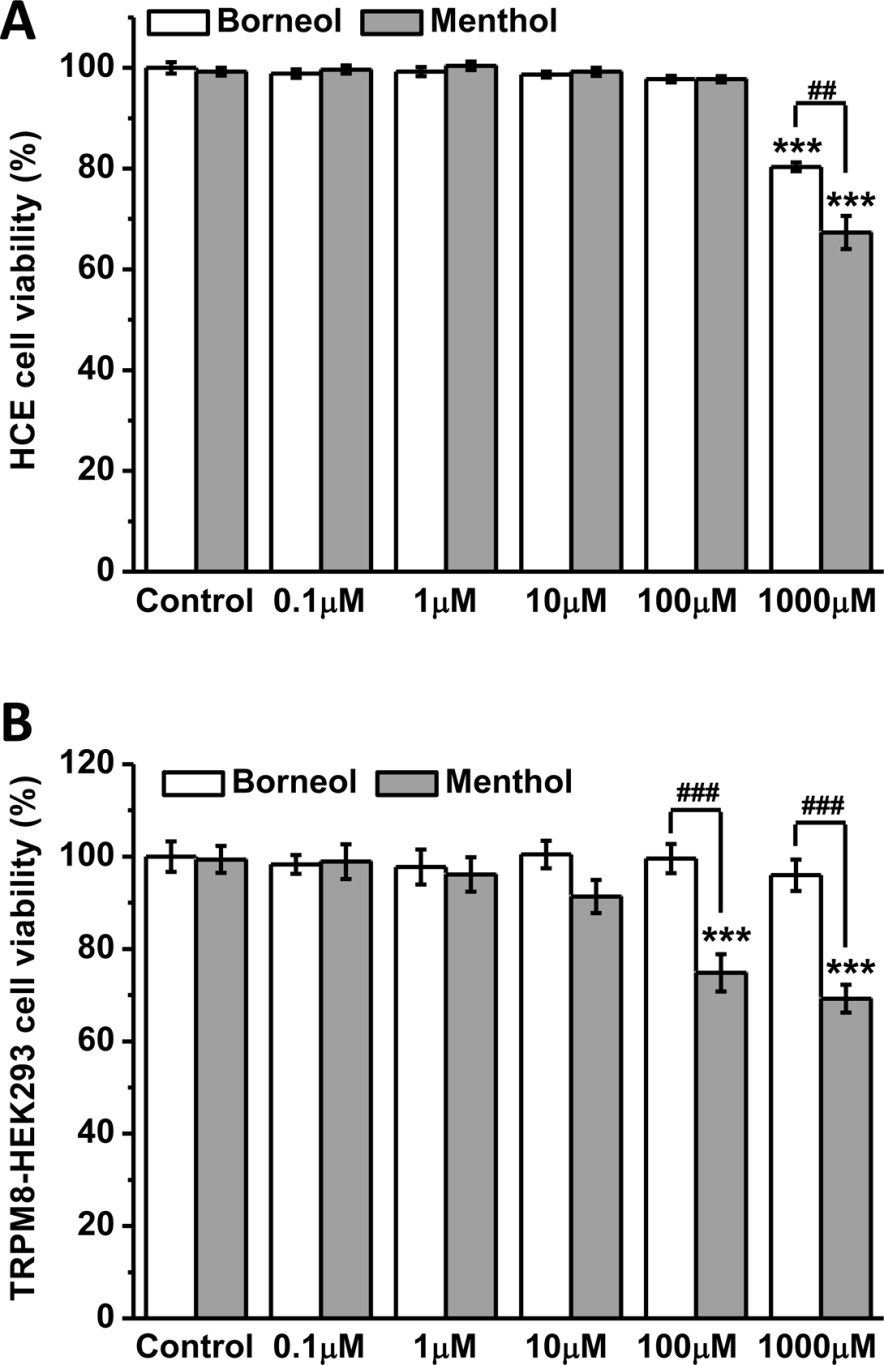
Effects of borneol and menthol on viability of human corneal epithelial (HCE) cells and TRPM8-expressing HEK293 cells. (A) Both borneol and menthol at 1 mM significantly reduced the viability of HCE cells. (B) Borneol at 0.1–1000 μM did not affect the viability of TRPM8-HEK293 cells, while menthol at 100 and 1000 μM showed strong inhibition on cell viability. (*** *P*<0.001 *vs* control; ^##^ *P*<0.01, ^###^ *P*<0.001 between indicated columns; *n* = 8 in each column).

We used WST-1 assay to assess the impact of borneol and menthol on the viability of human corneal epithelial (HCE) cells and TRPM8-expressing HEK293 cells. After incubation with the drugs for 24 h, the viability of HCE cells was not affected by borneol and menthol at 0.1–100 μM, while both drugs at 1 mM significantly inhibited the growth of the cells (Fig 5A). The inhibitory effect of borneol was weaker than that of menthol, suggesting a lower toxicity of borneol to corneal epithelium. For TRPM8-HEK293 cells, borneol at 0.1–1000 μM showed no effect on cell viability. In contrast, menthol at 100 and 1000 μM strongly reduced the viability of the cells (Fig 5B). These results indicate that at the same concentration as borneol, menthol is prone to induce intracellular Ca^2+^ overload and cell death mediated by TRPM8 channel.

## Discussion

Borneol has long been used in China with other herbal extracts to treat oculopathies, including dry eye syndrome [29]. The treatment with borneol and other compounds produced beneficial effects to the patients [26], but the contribution of each component and the therapeutic mechanism are unclear. In the present study we demonstrated that micromolar borneol can significantly increase lacrimation in guinea pigs at 25°C but not 35°C, suggesting a temperature-sensitive target of borneol in the cornea. In addition, the TRPM8 channel blockers AMTB and BCTC dramatically inhibited tear secretion induced by borneol and menthol. In the measurement of nociceptive behaviors, the response to borneol was similar to that of menthol, which has been proved to be non-irritating for rodents at micromolar concentration [17]. These results strongly suggest the TRPM8-expressing afferent cool cells in the cornea are responsive to borneol, since these cells are the only type of neurons that trigger lacrimation without nociceptive response upon activation [17, 30]. The expression of TRPM8 in the cornea of guinea pig has been previously detected by Madrid et al. [18] and confirmed in current study. Using TRPM8-expressing HEK293 cells, we found that TRPM8 channel is indeed a pharmacological target of borneol, and the drug-channel interaction at the intracellular side leads to channel opening and Ca^2+^ influx into the cells. As that established in mice [16, 17] and rats [20], TRPM8-mediated Ca^2+^ signaling eventually triggers lacrimation through a neuroregulation pathway [21].

In addition to corneal nerve fibers, expression of TRPM8 has been detected in cultured human corneal epithelial (HCE) [31] and endothelial cells [32], corneal stromal keratocytes [33], and conjunctival epithelial cells [34]. We also examined the expression of TRPM8 in primary HCE cells. The results showed that the level of TRPM8 mRNA was very low, and TRPM8 protein in these cells was undetectable (S1 Fig). This is inconsistent with the previous study that demonstrated functional expression of TRPM8 in immortalized HCE cells [31], suggesting the expression of TRPM8 in HCE cells may be sensitive to viral infection and culture conditions. As the primary HCE cells we used have been transported overseas and proliferated *in vitro*, it will be more reliable to perform immunostaining for TRPM8 in intact cornea to examine the expression in different cell types. *In situ* distribution of TRPM8 in the cornea was observed by using transgenic mice carrying a GFP gene driven by the TRPM8 promotor [19]. The intensity of GFP in nerve fibers was much higher than that in corneal stroma and epithelium, suggesting higher expression of TRPM8 channel in corneal sensory neurons, so that action potential can be initiated by cold stimulus. Nonetheless, since the expression level may not be proportional to the functionality of channels, it would be interesting to investigate the current density of TRPM8 in corneal sensory neurons and surrounding non-excitable cells, and also the interplay of channels between excitable and non-excitable cells. The presence of TRPM8 in corneal epithelial cells may relate to corneal functions such as the permeability of corneal epithelium. As mentioned in the introduction section, a number of studies have demonstrated that borneal can increase corneal permeability and promote the penetration of other drugs through this barrier tissue. Activation of TRPM8 channels and other potential targets of borneol in corneal epithelial cells may be responsible for this effect. Borneol has been found to activate TRPV3 channel [35], which belongs to the heat-activated vanilloid family of TRP channels. The members of this family are expressed in corneal nerve fibers (TRPV1) [36], and epithelial [37, 38] and endothelial cells (TRPV1/2/3/4) [39, 40]. TRPV1 is well known as the receptor for capsaicin [41], the pungent ingredient of chilli pepper that induces strong nociceptive responses upon eye contact. Considering the wide use of borneol-containing eye drops in China, and our finding that borneol did not cause nociceptive response in guinea pigs, it can be deduced that borneol is unlikely to excite TRPV1-expressing nociceptive sensory neurons in the cornea. In corneal epithelium, TRPV1 plays a pro-inflammatory role [42, 43] and is involved in wound healing by regulating proliferation and migration of epithelial cells [44]. TRPV4 is required for regulatory cell volume decrease in response to hypotonic challenge [45]. Although the expression of temperature-sensitive TRP channels in the cornea has been demonstrated in recent years, the functions of these channels are still largely unknown, except their general role as temperature sensors [39, 46]. To better understand the ocular pharmacology of borneol, further studies to test the effects of borneol on TRPV channels are required.

Borneol is categorized as an ocular irritant in the Material Safety Data from chemical providers such as Fisher Scientific and Sigma-Aldrich. It is suggested to rinse the eyes immediately upon contact to borneol. This is contradictory with the popular use of borneol-containing eye drops in China. The production of eye drops and other ophthalmic preparations is critically controlled by China Food and Drug Administration. It is unlikely that a compound with acute toxicity could be used in OTC drugs in such a wide range for decades. Toxicological studies in rabbits demonstrated that frequent corneal application (4 times/day for 1 month) of borneol at concentrations up to 1 g/L (6.48 mM) did not harm the eyes and other organs including liver, spleen and kidney [47, 48]. Acute irritating response (eye closure) was observed only in the group treated with 1 g/L borneol [47]. Another study found that 1 g/L borneol neither irritated the eyes nor damaged the corneal epithelium of rabbits [7]. Our result in guinea pigs suggests that micromolar borneol is not irritative to the animals, which is consistent with the conclusion from above studies in rabbits. According to a survey on borneol-containing ophthalmic preparations, the concentrations of borneol in the eye drops are generally in the range of 0.001–100 g/L [49]. This raises a concern about the risk of borneol overdose when some of these eye drops are used. Since most eye drops are mixtures of several compounds, the potential irritating effect of high-dose borneol may have been masked or attenuated by other components. For safety, it is strongly suggested to reduce the dose of borneol in ophthalmic preparations to less than 1 g/L. The ocular toxicity of borneol should be further examined to critically evaluate the risk of overdose in eye drops.

The effect of borneol on TRPM8 channel was first and briefly examined by Vogt-Eisele et al. [35]. The authors found that 2 mM borneol was not sufficient to potentiate large currents in TRPM8-expressing HEK293 cells. In the present study we performed Ca^2+^ imaging and patch clamp experiments on the same type of cells. The results from both experiments came to the same conclusion that borneol is indeed an activator of TRPM8 channel, though the potency is much lower than menthol. In the previous study Vogt-Eisele et al. [35] used a voltage clamp protocol holding at -40 mV, and the amplitudes of TRPM8 currents induced by 2 mM borneol were less than 10% of that induced by 2 mM menthol. We used the same protocol on our cells and found that the currents potentiated by 2 mM borneol accounted for ~60% of that by 2 mM menthol (S3 Fig). Compared to the relatively small menthol-induced current (~0.7 nA) in the previous study, the currents we recorded were always larger (1.4–2.0 nA). This suggests that the expression level of TRPM8 in our stably transfected cells may be higher than that used in the previous study. For drug testing on TRPM8 channel, these cells may be particularly useful for compounds with relatively lower efficacy. Taking this advantage, we were able to demonstrate the concentration-dependent activation of TRPM8 channel by borneol. However, as the highest concentration we tested (2 mM) is very close to solubility limit of borneol in the standard bath solution (diluted from 2 M stock in DMSO), the EC_50_ was not calculated due to the lack of maximum activation data. Higher concentrations of borneol in eye drops are achieved by using cosolvent mixed with ethanol, phenoxyethanol and polyvinyl alcohol, and adding surfactant tween-80 and solubilizing agents such as sodium citrate. Therefore, the activation of corneal TRPM8 channels and induction of tearing response by borneol could be more profound when these eye drops are used.

We conclude that borneol can increase tear secretion via activating the cold-sensing TRPM8 channel in the cornea, which is a novel therapeutic mechanism of borneol-containing ophthalmic preparations.

## Supporting Information

S1 Fig Detection of TRPM8 expression in HEK293 and human corneal epithelial cells. Western blot (A) and RT-PCR (B) of HEK293 cells transfected with TRPM8 cDNA plasmids (lane 1), mock transfected HEK293 cells (lane 2), primary human corneal epithelial cells (lane 3) and no-RT control (lane 4). Rabbit polyclonal anti-TRPM8 primary antibody (D122681, Sangon, Shanghai, China) was used at 1:500 dilution. The expected size of TRPM8 protein is ~120kDa.
Primers:
TRPM8-F: 5’-CAATGCCATCTCCTACGCTC-3’
TRPM8-R: 5’-CAGCAGGAGGAAGGCGATGTAG-3’ (product size: 1039bp)
beta-actin-F: 5’-ACAGAGCCTCGCCTTTGC-3’
beta-actin-R: 5’-GGAATCCTTCTGACCCATGC-3’ (product size: 211bp).
(TIF)

S2 Fig Inhibition of borneol-induced TRPM8 activity by BCTC. (A) Ca^2+^ imaging. (B) Whole-cell patch clamp.
(TIF)

S3 Fig Comparison of whole-cell currents induced by 2 mM menthol and borneol in TRPM8-expressing HEK293 cells with voltage holding at -40 mV. (A) Example recording. The interval time for washout is 2 min. (B) Mean±SD of currents induced by 2 mM menthol and borneol (*n* = 4).
(TIF)
