# Supplementary figures and images for: Borneol Is a TRPM8 Agonist that Increases Ocular Surface Wetness
Source: PLoS One. 2016 Jul 22;11(7):e0158868. doi: 10.1371/journal.pone.0158868 (PMC4957794; doi:10.1371/journal.pone.0158868)

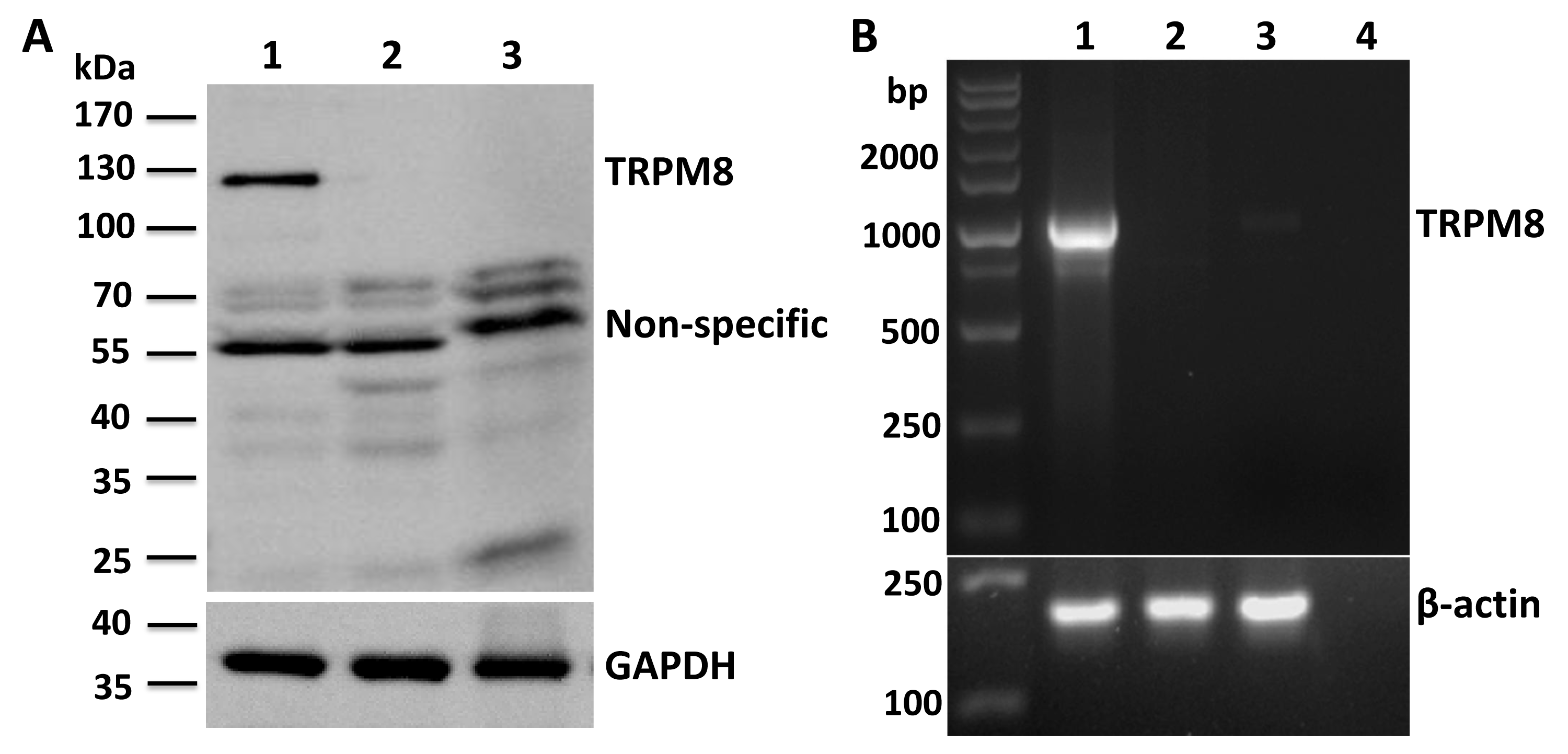

Supplement: S1 Fig — Western blot (A) and RT-PCR (B) of HEK293 cells transfected with TRPM8 cDNA plasmids (lane 1), mock transfected HEK293 cells (lane 2), primary human corneal epithelial cells (lane 3) and no-RT control (lane 4). Rabbit polyclonal anti-TRPM8 primary antibody (D122681, Sangon, Shanghai, China) was used at 1:500 dilution. The expected size of TRPM8 protein is ~120kDa. Primers: TRPM8-F: 5’-CAATGCCATCTCCTACGCTC-3’ TRPM8-R: 5’-CAGCAGGAGGAAGGCGATGTAG-3’ (product size: 1039bp) beta-actin-F: 5’-ACAGAGCCTCGCCTTTGC-3’ beta-actin-R: 5’-GGAATCCTTCTGACCCATGC-3’ (product size: 211bp). (TIF) [file pone.0158868.s001.tif]

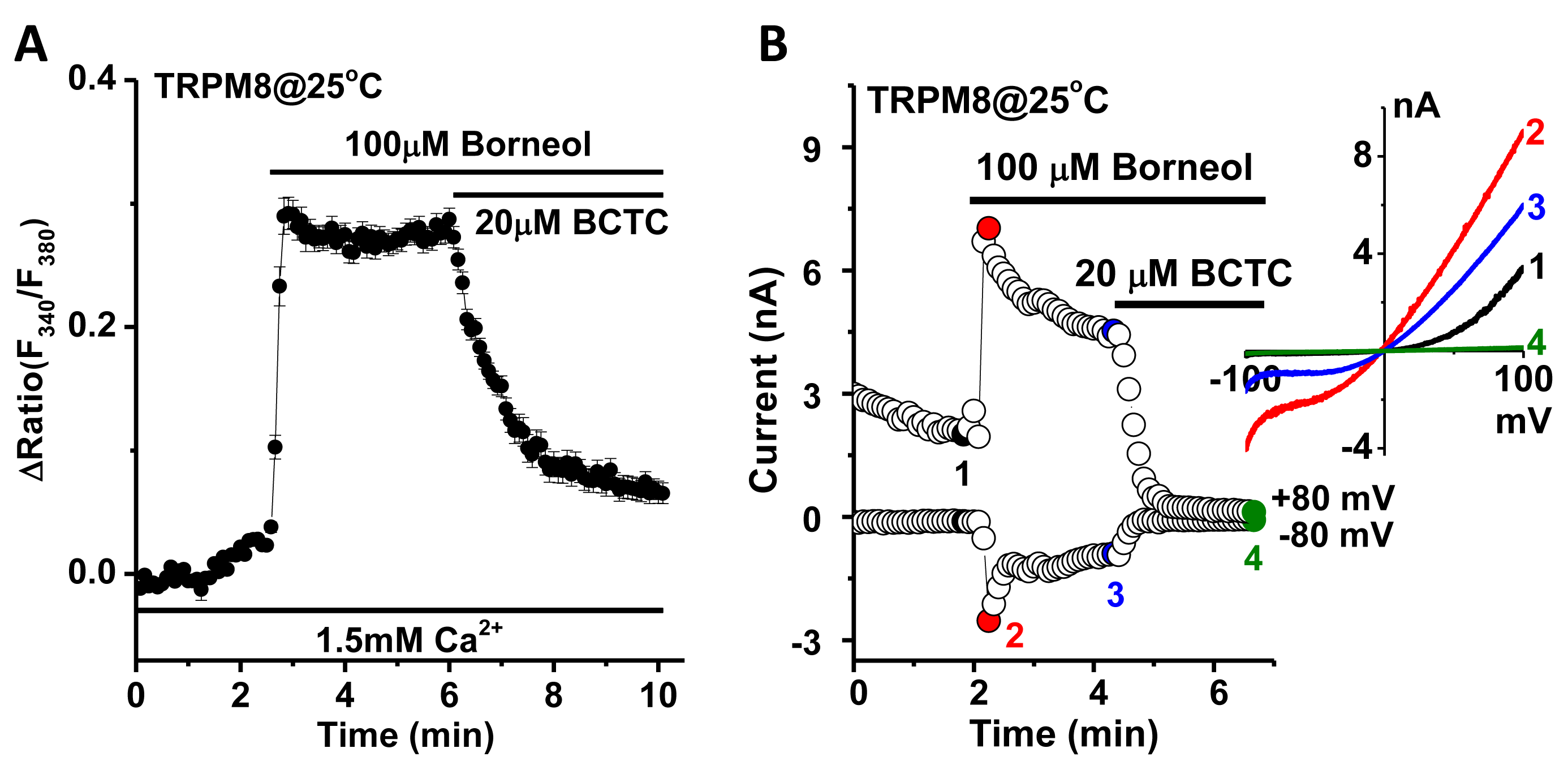

Supplement: S2 Fig — (A) Ca2+ imaging. (B) Whole-cell patch clamp. (TIF) [file pone.0158868.s002.tif]

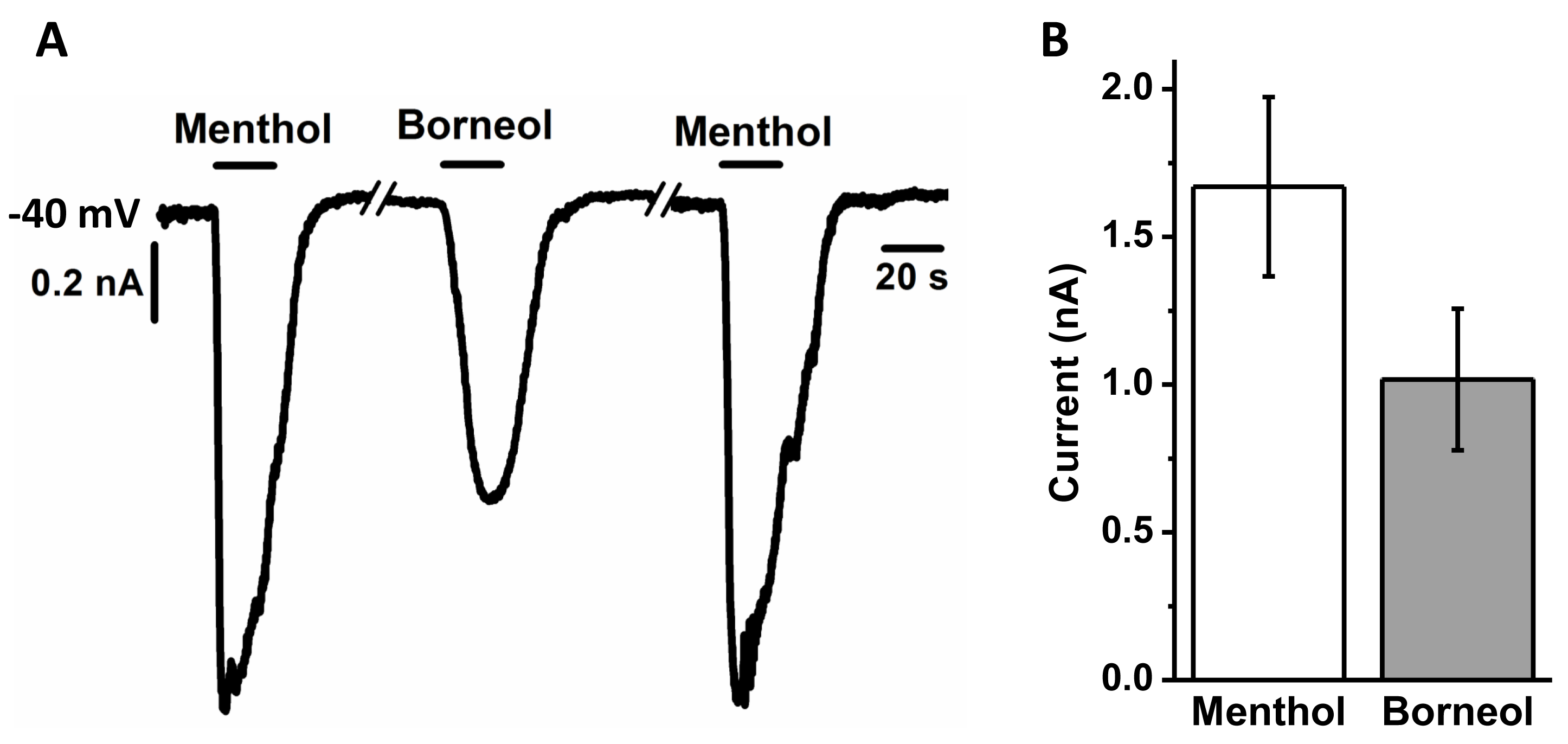

Supplement: S3 Fig — (A) Example recording. The interval time for washout is 2 min. (B) Mean±SD of currents induced by 2 mM menthol and borneol (n = 4). (TIF) [file pone.0158868.s003.tif]
